# National strategies for screening neural tube defects in Saudi Arabia: activating prevention and early intervention

**DOI:** 10.3389/fpubh.2025.1507446

**Published:** 2025-09-19

**Authors:** Mariam AlEissa, Wejdan Hakami, Andreas Dimopoulos, Saleha Abdul Rab, Noor Alsaadoun, Zohaib Asim, Cham Jazieh, Khadijah Ateq, Noara Alhusseini

**Affiliations:** ^1^Department of Epidemiology and Biostatistics, College of Medicine, Alfaisal University, Riyadh, Saudi Arabia; ^2^Public Health Lab, Public Health Authority (Saudi Arabia), Riyadh, Saudi Arabia; ^3^Research Department, King Khaled Eye Specialist Hospital, Riyadh, Saudi Arabia; ^4^Computational Sciences Department at the Centre for Genomic Medicine (CGM), King Faisal Specialist Hospital and Research Centre, Riyadh, Saudi Arabia; ^5^Division of Pediatric Neurology, Department of Pediatrics, Prince Sultan Military Medical City, Riyadh, Saudi Arabia; ^6^College of Law, Alfaisal University, Riyadh, Saudi Arabia; ^7^Research Office, King Abdullah International Medical Research Center (KAIMRC), Riyadh, Saudi Arabia

**Keywords:** neural tube defect, NTD, genetic screening, food fortification, Saudi Arabia

## Abstract

**Background:**

Neural tube defects (NTDs) are serious congenital anomalies affecting the brain and spinal cord. Despite widespread folic acid supplementation and food fortification programs, regions such as Saudi Arabia have not experienced a proportional decline in NTD prevalence. This narrative review evaluates the multifactorial contributors to NTDs, focusing on the effectiveness of current prevention and screening strategies both globally and within Saudi Arabia.

**Materials and methods:**

A comparative methodology guided this review, drawing from studies published between 2000 and 2024 sourced from PubMed, Scopus, and the WHO library. Keywords included “neural tube defects,” “folic acid supplementation,” “screening programs,” and “food fortification.” While not a systematic review, PRISMA principles were loosely followed to ensure study relevance and rigor.

**Results:**

Globally, countries like the United States, Canada, Chile, and Australia have implemented mandatory folic acid fortification and reported NTD reductions ranging from 19 to 78%. South Africa, for example, achieved a 66% decline in NTD-related deaths post-fortification. In Saudi Arabia, similar initiatives have been launched, including folic acid campaigns and food fortification. However, national-level data evaluating their impact remains sparse. Regional disparities in implementation, awareness, and access have limited the success of these measures. Although 80.1% of Saudi women reportedly understand the preventive role of folic acid, uptake and proper timing of supplementation remain inconsistent. Screening services, particularly in rural areas, are not uniformly accessible, reducing early detection rates. Unlike countries such as Australia and Chile, Saudi Arabia lacks a standardized system for tracking and evaluating NTD outcomes.

**Conclusion:**

This review concludes that while Saudi Arabia has adopted commendable preventive strategies, the absence of comprehensive data, policy enforcement, and public education limits their effectiveness. Strengthening national monitoring systems, ensuring equitable access to screening, and enforcing mandatory fortification policies modelled on successful international practices are critical. Adopting evidence-based policies supported by robust evaluation frameworks will be essential to reducing the burden of NTDs and improving maternal and child health outcomes in Saudi Arabia.

## Background

Neural tube defects are prevalent birth anomalies caused by early developmental abnormalities in the formation of the brain and spinal cord (1, 2). These defects happen between 21 and 28 days after conception when the neural folds fail to join in the middle to form the neural tube (3, 4). Key neural tube defects include spina bifida, meningocele, myelomeningocele, encephalocele, anencephaly, caudal regression syndrome, tethered cord, and syringomyelia (5). The severity of motor, sensory impairments, and incontinence in NTDs depends on the level of damage. It can also be linked to brain issues like Chiari II malformation, hydrocephalus, abnormal brain cell positioning, and underdevelopment of structures like the corpus callosum and cranial nerve centers (6). Children with NTDs require ongoing care from a team of experts. This team should include specialists in neonatology, pediatric neurology, neurosurgery, urology, pediatric orthopedics, and physical medicine (7). These conditions not only lead to significant morbidity and mortality but also cause immense psychological and financial stress on families and communities (8).

### Risk factors

Several factors, including nutrition, increase the risk of NTDs. A key risk is a lack of folate (B9) in the mother before and during early pregnancy (9). Research shows that low folate levels, measured in red blood cells, are a major cause of NTDs (7, 10). Studies show that a 5–6 per 10,000 live births NTD rate is achieved in countries with successful large-scale folic acid food fortification programs (11). Vitamins B2, B6, B12, choline, betaine, and n-3 fatty acids can lower NTD risk by affecting the one-carbon metabolism pathway (12). Genetic factors, such as mutations in the methylenetetrahydrofolate reductase (MTHFR) gene, increase the risk of NTDs (13). Maternal folate receptor antibodies, which block folate transfer from mother to fetus, can disrupt folate transport and affect neural tube development, increasing the risk of NTDs (14). Infectious diseases like malaria and certain drugs, including thalidomide, methotrexate, anti-seizure medications, and some antimalarials, increase the risk of NTDs (7). Metabolic disorders like diabetes before pregnancy and phenylketonuria can also cause NTDs (15). Exposure to chemicals like solvents, arsenic, pesticides, paints, radiation, and toxic metals such as mercury and lead increases the risk of NTDs (7). Both a lack of manganese and too much manganese can be harmful to the fetus (16).

### Method

This review presents a narrative synthesis of literature retrieved from PubMed, Scopus, and the WHO databases, following PRISMA guidelines. Predefined keywords were utilized to narrow the search to NTD, screening, folic acid, and policy. Inclusion for the paper selected was limited to articles published between 2000 and 2024 and peer-reviewed in English. This paper focuses on raising folate levels in the population to lower the risk of NTDs. It will:

1. Analyze the issue using the most recent global data on folate levels in women of reproductive age and rates of NTDs
2. Summarize current interventions and identify areas for improvement.

Suggest policies that a nation could include in its strategic plan for controlling folate insufficiency and folic acid-responsive neural tube defects, ultimately leading to improved health outcomes for women and children

## Results

### The impact of NTD on public health

#### NTDs’ global and local burden on healthcare

NTDs are a major cause of infant death and disability, affecting around 300,000 newborns each year worldwide, with rates as high as 199.4 per 10,000 births. These conditions possess a public health issue of global significance, with prevalence rates that seem uninfluenced by a country’s economic status or level of development (15, 17, 18).

#### Folate status estimation

To determine one’s folate status, the most often utilized indicators are RBC and serum/plasma folate levels. Serum or plasma folate shows short-term folic acid intake, while red blood cell folate reflects long-term consumption (7, 19, 20). When assessing the folate status of a population, the RBC folate level is preferable. The WHO advocates measuring RBC folate using a harmonized Microbiological Assay (MBA) to obtain consistent, dependable findings throughout time and regions (7, 10). A deficiency in folate raises the risk of megaloblastic anemia, with serum folate levels below 7 nmol/L and RBC folate levels below 227 nmol/L serving as the cut-off thresholds (7, 10). Increased cut-off levels are necessary to offer optimal protection against folate-dependent NTDs because higher folate concentrations are required to promote immediate cell division at neural tube closure (7). Folate deficiency is defined as having folate levels below this threshold. The WHO recommends an RBC folate level of 906 nmol/L (or 748 nmol/L with a different calibrator) as the key protective level against NTDs. Folate levels are directly related to the risk of NTDs (7, 19, 20).

#### Folate levels in women of childbearing age

Worldwide, data are scarce addressing folate status. A recent systematic analysis listed every country-specific survey published between 2000 and 2014 that indicated a global folate deficiency or insufficiency. Over 70% of the accessible data showed high- or upper-middle-income nations. The majority of nations had greater than 40% prevalence of folate insufficiency. This review exposed many gaps in our knowledge of folate status all over the world. They were lacking in information about folate insufficiency, heterogeneous laboratory methods used (only 10 surveys measured RBC folate using the recommended harmonized MBA), and a scarcity of data [only 39 countries completed 45 surveys total for this evaluation during the study period (21)].

#### NTD’S prevalence

The prevalence of NTD has proven to be challenging to estimate and frequently imprecise, underestimating the actual value. There are several reasons for this challenge; for example, the majority of countries lack adequate birth defect surveillance systems that include data on stillbirths and abortions. Most recent estimates of NTD prevalence come from a thorough study by Blencowe et al. (22), which determined global prevalence based on published literature and national birth registries. In 2015, there were about 260,100 new NTD cases worldwide, with 117,900 (75%) leading to death in children under five, mostly in low- and middle-income countries (LMIC). The authors reported these NTD rates per 10,000 live births without folic acid fortification: Sub-Saharan Africa (15.27), Southern Asia (31.96), East Asia (19.44), and Northern Africa and Western Asia (17.45) (22).

In Saudi Arabia, NTDs occur in about 1 in 1,000 births (23). Although this is lower than the global average of 1 in 1,500 births, it is higher than in some developed countries, like the United States, where the rate is 1 in 2,500 births (24–26), Table 1. Uncovering the factors behind this regional discrepancy is crucial for targeted public health interventions.

**Table 1:** Fortification policy and impact: comparative overview of neural tube defect prevention strategies across select countries.

Country	Fortification policy	NTD prevalence (Before/After)		Policy features
USA (48)	Mandatory since 1998.	~10 per 10,000	~7 per 10,000	Broad national coverage, early implementation
Canada (31)	Mandatory	~14 per 10,000	~7.6 per 10,000	Strong monitoring and enforcement
Chile (34)	Mandatory wheat flour fortification	~17 per 10,000	~10 per 10,000	Regional campaigns supported by legislation
Costa Rica (37)	Mandatory	~15 per 10,000	~8 per 10,000	Integrated surveillance and health education
South Africa (42)	Fortification of maize and wheat	~11 per 10,000	~3.7 per 10,000	Cost–benefit justified, strong rural outreach
Australia (46, 97)	Mandatory fortification (2009)	~12.8 per 10,000	~8.7 per 10,000	Significant drop after 2009; national monitoring
Japan (98)	Voluntary folic acid fortification	~6 per 10,000	~5.9 per 10,000	Minimal reduction; fortification not mandatory
Saudi Arabia (23, 24)	Fortification + awareness campaigns	~15 per 10,000	~10 per 10,000	Mixed coverage, knowledge-use gap, urban-focused

#### Economic status

Neural tube defects (NTDs) can have significant economic impacts on affected individuals, families, and society. The financial cost of NTDs is high. The medical expenses associated with diagnosing, treating, and managing NTDs can be substantial (27, 28). This includes expenses related to prenatal screening, diagnostic tests (such as ultrasound and amniocentesis), surgeries (such as corrective procedures for spinal defects), hospitalizations, medications, and ongoing medical care. It may also include physical, speech, and occupational therapy, mobility aids, assistive devices, and other treatments for physical and cognitive needs (27). In the USA, the estimated annual medical costs per patient with NTD were ($51,574 in 2003), and for spina bifida, they ranged from ($11,061 in 1993) to ($65,177 in 2003). For treating patients with spina bifida, the Spanish Social Security system paid medical costs of ($2,734 or $2,953,138 in 1988) per year per person annually. This financial burden would decrease if the incidence and prevalence of NTDs decreased (29). Countries with higher income levels typically have more prevalent systems for monitoring neural tube defects. There was a lack of data from countries in Africa and Southeast Asia (30).

#### Large-scale food fortification (LSFF)

Many countries have made folic acid fortification in staple foods mandatory because it helps reduce the risk of NTDs in pregnancies (31). Programs in China, the U. S., Canada, Costa Rica, South Africa, and Chile have shown that folic acid can reduce NTD rates to as low as 5–6 per 10,000 pregnancies (31). A study showed food fortification is generally cost-effective, with a return of 17.5:1 for each monetary unit spent (32). Large-scale food fortification (LSFF) aims to improve public health by boosting nutrient intake without changing regular food habits (32). In 2023, the WHO adopted a plan to accelerate efforts to prevent micronutrient deficiencies and NTDs through food fortification, urging countries to base decisions on public health needs and regular monitoring (33).

When implemented properly by governments, large-scale food fortification (LSFF) can significantly improve public nutrition (34). Research is needed to fully understand its health impacts (34). LSFF combined with folic acid has successfully increased folate levels in women of reproductive age and reduced the prevalence of NTDs, particularly in low- and middle-income countries (LMICs) (35). Countries like Costa Rica, Brazil, Mexico, and South Africa have seen reductions in NTDs by 30–59% after fortifying foods like wheat, maize, and rice with folic acid (36–43). However, despite mandatory supplementation, China still has a high NTD prevalence (44).

Australia’s experience shows that NTD rates dropped significantly after mandatory fortification was introduced in 2009 (45, 46). Before that, only voluntary fortification had a smaller impact (46). In the U.S., mandatory folic acid fortification began in 1998 to ensure women of childbearing age received adequate folate, helping reduce NTD rates (47). However, many countries, especially in Asia and Europe, still lack mandatory folic acid fortification, leading to higher rates of spina bifida (48). Data from South Africa, Argentina, and Costa Rica show a significant reduction in NTD-related deaths following mandatory fortification, with decreases of 66, 68, and 71%, respectively (38, 39, 42, 48).

## Discussion

### Health interventions and strategies for prevention

#### Variation in diet

Folate is present in foods such as leafy green vegetables, beans, lentils, broccoli, avocado, nuts, seeds, fruits, and animal products like eggs and liver. It can be hard to get the recommended daily folate from food alone, though nutrition education should highlight folate-rich foods and their benefits. Current folic acid guidelines include the folate from a healthy diet (49).

#### Oral folic acid supplementation for periconceptional primary prevention

Women planning a pregnancy are advised by the CDC, Institute of Medicine, and US Preventive Services Task Force to take 400–800 μg of folic acid daily, beginning 4 weeks before conception and continuing through the first trimester (50). A systematic review of clinical trials involving 7,391 women found that folate supplements taken before conception can prevent up to 70% of NTDs (51, 52). Women taking anticonvulsants or those with diabetes are recommended to take 5,000 μg of folic acid daily, along with adhering to dietary guidelines (49). A recent review found that folic acid fortification and supplementation programs led by governments effectively reduced NTDs. They also emphasized the importance of education and following recommendations (31). Unplanned pregnancies are higher in low- and middle-income countries compared to High-Income Countries (HIC) (53). Access to prenatal care may be unequal, with wealthier, more educated women, or those with a history of difficult pregnancies, more likely to seek it (54, 55). In China, preconception folic acid use has helped decrease NTD rates from 2000 to 2017 (29). However, supplementation does not effectively prevent unplanned pregnancies (53).

#### Oral folic acid supplementation to prevent recurring issues before conception

NTD recurrence means a neural tube defect happens again in a future pregnancy after it occurred in a previous one. To prevent recurrence, a daily intake of 4,000 μg of folic acid, starting 1 month prior to a planned pregnancy and continuing through the first trimester, has been highly recommended (49, 56, 57). A systematic study of pooled data from Great Britain, North America, and Europe finds that there is a 4% chance of recurrence after one NTD-affected pregnancy and an 11.1% risk after two previously affected pregnancies (51). Four randomized trials investigating folic acid supplementation for preventing NTD recurrences demonstrated an NTD rate of 0.6% among those taking the supplement, compared to 4.1% in those who did not, indicating an 87% reduction in risk (58).

#### Weekly supplementation of folic acid

Currently, no randomized trials evaluate the effectiveness of weekly periconceptional folate supplementation in preventing neural tube defects (NTDs). However, three studies found that weekly folic acid improved folate levels, with one showing reduced NTD rates (7). In one trial, 74 women (39 with prior NTD pregnancies) received 5,000 μg of folic acid weekly for 3 months, leading to significant increases in plasma and RBC folate in 90% of participants. Based on these results, a program provided free supplements to 250,000 low-income women, reducing NTD rates from 10.04 to 5.8 per 10,000 live births over 28 months (7). Another study included 114 women compared weekly doses of 2,800 μg of folic acid, daily 400 μg doses, and a placebo. It found that 49% of the weekly group had a protective increase in folate levels, but this was lower than the 74% in the daily 400 μg group (58). Another study with 331 young women (average age 18) showed that weekly 2,800 μg doses raised RBC folate levels more than 400 μg or no supplementation (59). Women in the 2,800 μg group were also seven times more likely to reach protective folate levels (RR = 7.3, 95% CI: 3.9–13.7) (59).

#### Educational campaigns about folic acid supplementation

Many women do not get enough folic acid due to a lack of awareness about its role in preventing NTDs. Prenatal counseling can help those planning pregnancies but is often overlooked (60, 61). Most women learn they are pregnant around 5 weeks, but by then, it’s too late to start taking folic acid since the neural tube closes within 4 weeks (61). From 2010 to 2014, 44% of pregnancies worldwide were unplanned, with even fewer planned in teens and women in LMICs, with limited information (61). In 2015, a Safe food campaign increased awareness of folic acid from 27.5 to 60% and supplement use in women not planning pregnancy from 13 to 17% (62).

Awareness campaigns about taking folic acid during pregnancy have been successful (62). However, supplement use is still low, even among women planning pregnancies (63). A study conducted in the U.S. and Canada found that only 13% of women with planned pregnancies used folic acid before conception, despite 60% planning their pregnancies (63). Similarly, high-income countries show gaps in usage: 3%/77% in the UK, 14%/76% in Denmark, and 31%/80% in Norway (64–66). Barriers include young age, ethnicity, and low socioeconomic status (67). Campaigns relying on printed materials or media had limited long-term impact, but healthcare-based efforts could work if supplements are accessible, especially for vulnerable women (67).

**Figure 1:**
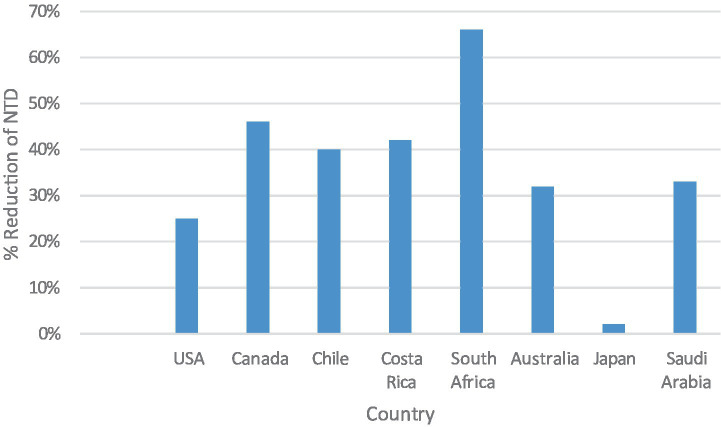
Percentage reduction in neural tube defect (NTD) prevalence following folic acid fortification across various countries.

In evolving and high-income countries such as the United States and Canada, the prevalence of neural tube defects (NTDs) reduced after introducing a mandatory folic acid fortification policy in the late 1990s led to substantial declines in reported reductions, for example in the United States the prevalence was 19–32% and up to 46% in Canada (31, 48). Case reductions post-fortification were observed in Costa Rica and Chile, achieving 35–49% (34) as shown in Figure 1. The difference in program implementation justifies the suggested regional variation in program coverage, population-level adherence, or baseline folate deficiency. The Saudi government implemented both food fortification and supplementation campaigns, resulting in a decrease from 1.5 to 1.0 per 1,000 live births between 2000 and 2020 (23, 24).

#### Health interventions and strategies for prevention: a focus on Saudi Arabia

Various health interventions have been introduced worldwide and in Saudi Arabia to prevent NTDs, including ultrasound, prenatal screening, and adding 400 g of folic acid to the daily diet, which has greatly reduced NTDs in newborns (68–71). However, the recent decline in NTD rates in Saudi Arabia is thought to be due to several different factors (70). A key factor in reducing NTDs in Saudi Arabia is widespread folic acid supplementation among women of reproductive age (70). Additionally, fortification programs have shown significant reductions in NTD rates (70). The country’s young population also helps, as women under 25 are generally at a lower risk of having babies with NTDs compared to women over 35 (71). Mothers under 25 years face a slightly increased risk of NTDs due to behavioral factors or modifiable nutritional not biological age. These factors include low folic acid intake, unplanned pregnancies, inadequate diets, and micronutrient deficiencies, e.g., iron and vitamin B12. Further Socioeconomic challenges, education, and limited healthcare access compound these risks (22, 72–74), while women over 35 have a higher risk of NTDs due to age-related metabolic instability and comorbidities like obesity and diabetes (73). Represented by the MOH, the government of Saudi Arabia has worked through the years on several initiatives to prevent NTDs, as illustrated in the timeline summary in Table 2 (75–83). The Saudi Ministry of Health (MoH) has worked hard to raise awareness about folic acid supplementation. Their website provides clear guidelines on daily folic acid intake: 400 μg for normal-risk women before and during pregnancy (up to 12 weeks) and 5 mg for high-risk women (such as those on antiepileptic or antidiabetic drugs, or with a history of neural tube defects). A study by Alreshidi et al. found that 67.5% of pregnant women in Saudi Arabia received information on folic acid from their healthcare provider, and 80.1% were aware that it prevents NTDs (84). The MOH has also introduced prenatal ultrasound screening and a strategy to fortify specific foods with folic acid (77, 85). Genetic counseling is offered to couples, given the region’s high rates of consanguinity (75). Additionally, regular training for healthcare workers helps improve their ability to diagnose and manage NTDs (86).

**Table 2:** Prevent neural tube defects (NTDs) National Health Interventions in Saudi Arabia.

Intervention/Status	Action implemented	Implementing Body	Year Initiated	Impact
Folic acid fortification/mandatory	Wheat flour fortified with folic acid to reduce NTD risk	Saudi Food and Drug Authority (SFDA) (1)	2001	NTD incidence dropped from 1.9 to 0.76 per 1,000 live births (2, 3)
Premarital screening program/mandatory	Mandatory premarital screening for thalassemia and sickle cell anemia, which often co-exist with the risk of NTD.	Ministry of Health (MoH) (4)	2004	200,000 couples are screened annually, and the test prevents high-risk marriages (5, 6)
Maternal health education campaigns	Nationwide campaigns promote folic acid intake before and during early pregnancy.	MoH and regional health authorities [the Saudi Health Council (7), the Public Health Authority (Weqaya) (8), and the Saudi Food and Drug Authority (SFDA) (1)]	Ongoing	Improved public awareness (9)
NTD risk screening for antenatal	Ultrasound and AFP (biochemical testing) for early antenatal care	Primary Healthcare Centers at MoH	2010	Allows early prevention and intervention planning (10)

#### Screening initiatives for neural tube defects (NTDs) and their importance in Saudi Arabia

The screening for NTDs serves multiple crucial purposes. Primarily, early detection, especially of severe NTDs like anencephaly, furnishes healthcare professionals with the requisite knowledge to preempt potential complications during childbirth and the subsequent postnatal period, thereby enhancing the prognosis (87, 88). Furthermore, an early diagnosis in the gestation period equips expectant parents with the autonomy to make informed decisions (88). This could pertain to possible medical interventions or, more profoundly, considerations related to the prospective quality of life for the child (88). In a broader context, these screening programs offer a treasure trove of data. Such data aids researchers in delving deeper into the prevalence of NTDs, postulating potential etiological factors, and gauging the efficacy of preventive measures, like folic acid supplementation (89). From a fiscal standpoint, these screenings, when systematized, can prove to be cost-effective for the healthcare system (90). The ability to detect severe NTDs early can mitigate the substantial costs associated with extended hospital stays and complex surgical procedures (90). Congenital anomalies, particularly spinal cord anomalies like cystic dilatation of the ventriculus terminalis (CDVT), usually remain undiagnosed until later in adulthood. Emerging classifications underscore that most cases do not require surgical intervention, reinforcing the importance of ongoing clinical monitoring over immediate intervention or treatment (91).

#### Complexity and regulatory insufficiencies in screening measures for neural tube defects research in Saudi Arabia

Saudi Arabia operates two national screening initiatives primarily overseen by the Saudi Ministry of Health and various other government healthcare establishments (92). These programs consist of the premarital screening program and the National Newborn Screening Program (92). These initiatives have been implemented throughout the country, including detailed guidelines specifying the groups to be screened, the screening test locations, and the screening procedures. It is worth mentioning that both programs primarily focus on identifying genetic disorders (93). They either aim to identify individuals who are carriers of these disorders or those who are affected by them before they get married, or they work to detect affected newborns in their early stages (85). Dealing with the distinctive ethical dilemmas linked to screening measures and NTD-related research requires the involvement of experts to ensure appropriate oversight. In this context, the continued prenatal management of NTDs places parents in the challenging position of deciding whether to undergo fetal karyotyping and whether to proceed with or terminate the pregnancy (86). These decisions can give rise to significant ethical dilemmas, as they involve complex considerations related to the potential outcomes, personal values, cultural beliefs, and medical options, all of which must be carefully weighed to make informed choices. In the Saudi Arabian context, these ethical complexities surrounding Neural Tube Defects (NTDs) and prenatal screening are further heightened due to the presence of deeply ingrained socio-cultural norms and religious beliefs. Saudi Arabia’s society is deeply rooted in Islamic values, where healthcare practices are often intertwined with religious and cultural considerations. Decisions regarding prenatal screening, fetal karyotyping, and the continuation or termination of a pregnancy are not only influenced by medical factors but also by the ethical and moral principles dictated by Islamic teachings (87). This intersection of medical science, culture, and religion presents a unique set of challenges as individuals and families navigate the ethical dimensions of these decisions while adhering to their faith and cultural traditions. Moreover, within the Saudi healthcare system, existing ethical review boards predominantly adhere to a generalized scope of ethical oversight (87). These review boards evaluate the ethical aspects of medical research and healthcare practices (87). However, given the specialized nature of research domains such as screening measures and NTDs, this generalized approach may fall short of providing the nuanced scrutiny necessary to address the unique ethical considerations associated with these fields (87). In alignment with international ethical frameworks, the World Health Assembly is lobbying efforts to standardize screening frameworks for congenital anomalies, including NTDs. These efforts aim to promote global equity in access to screening and ensure ethical oversight in regulatory practices (94).

As a result, there is a growing need for developing specialized ethical guidelines and oversight mechanisms that specifically cater to the intricacies of NTD-related research and prenatal screening within the Saudi healthcare context. This would ensure that ethical decisions align with international standards and the cultural and religious values deeply embedded in Saudi society. The best solution is to ensure women of reproductive age get enough folic acid in a timely, effective, fair, and affordable way. Large-scale food fortification can accomplish all of these.

#### Alignment with global ethical standards and the international federation for spina bifida and hydrocephalus

An important ethical consideration in screening is aligning national regulations with global best practices. International guidelines like the Belmont Report, CIOMS, and the Helsinki Declaration provide frameworks for responsible research involving humans. The International Federation has also called for global action to reduce Neural Tube Defects (NTDs) and improve the rights of those with Spina Bifida and Hydrocephalus (SBH). However, preventing NTDs should not replace efforts to create inclusive societies for people with SBH. Funds for inclusion and accessibility should not be used for prevention programs. Prevention efforts must be designed carefully to avoid stigmatizing people with NTDs. Saudi Arabia, which ratified the UN Convention on the Rights of Persons with Disabilities (UNCRPD), must ensure that its NTD prevention policies respect the rights of people with SBH. Aligning with international ethical standards will improve the credibility and success of international partnerships, especially in multi-center studies on NTDs (95, 96).

#### Regulatory gaps in screening measures legislation

A glaring oversight in Saudi Arabia’s regulatory setting regarding screening measures for NTD research is the lack of comprehensive, specialized legislation. Despite significant advancements in healthcare and research-related legal frameworks, Saudi Arabian law requires updated provisions tailored to the complex nuances of screening measures and NTDs. Unlike more established areas of medical research, such as oncology, where guidelines are relatively mature, screening measures in the context of NTDs suffer from a noticeable absence of codified legal standards governing informed consent, data privacy, and biological sample ownership (77). Legislation should be developed and enforced by the Saudi Health Council (SHC, 2025), in collaboration with the National Committee of Bioethics (NCBE), the Public Health Authority (PHA, 2025), and the Saudi Food and Drug Authority (SFDA, 2025). To safeguard culturally appropriate, ethical, and legal principles, these institutions are best positioned to ensure alignment with international standards (99).

## Conclusion

Neural tube defects (NTDs) are serious birth defects that impact the brain and spinal cord’s development. They can be caused by several factors, with a lack of folic acid being the most well-known risk factor. Other factors include nutrition, genetics, maternal age, health conditions, environmental influences, toxins, medications, and socioeconomic status. The Kingdom of Saudi Arabia has implemented a comprehensive food fortification program that provides multiple dietary sources of supplemental folic acid and has recognized the importance of screening for NTDs. In 2019, the Ministry of Health launched a national screening program for NTDs. The program is offered to all pregnant women, and it includes a blood test and an ultrasound scan. The screening program has been successful in identifying NTDs early on. This has allowed for early intervention, which can improve the health outcomes for affected babies. However, there is still room for improvement. The screening program is not yet available in all parts of the country, and some women are not aware of it. The Saudi government needs to continue investing in screening programs and raising awareness about NTDs. By doing so, the country can help to prevent these serious birth defects and improve the health of its citizens. In conclusion, strategically implementing folic acid supplementation starting 1 month prior to a planned pregnancy and continuing through the first trimester is crucial, as this proven preventive measure can significantly reduce the incidence of neural tube defects. Additionally, the strategic implementation of screening initiatives for NTDs is pivotal in diminishing associated morbidity and mortality, thereby fostering an enhanced quality of life for affected individuals. Saudi Arabia’s comprehensive approach, which integrates early detection, public awareness, and preventive interventions, demonstrates its strong commitment to advancing maternal and child health.
